# Impact of Ultra-Processed Food Consumption on Quality of Diet among Brazilian Pregnant Women Assisted in Primary Health Care

**DOI:** 10.3390/ijerph20021015

**Published:** 2023-01-05

**Authors:** Walkyria O. Paula, Vivian S. S. Gonçalves, Erika S. O. Patriota, Sylvia C. C. Franceschini, Nathalia Pizato

**Affiliations:** 1 Graduate Program in Human Nutrition, Faculty of Health Sciences, University of Brasilia, Brasilia 70910-900, Brazil; 2 Graduate Program in Public Health, Faculty of Health Sciences, University of Brasilia, Brasilia 70910-900, Brazil; 3 Graduate Program in Nutrition Sciences, Federal University of Viçosa, Viçosa 36570-999, Brazil

**Keywords:** ultra-processed food, pregnancy, quality of diet, nutrients, primary health care

## Abstract

The quality of diet and nutritional status during pregnancy are crucial to optimize maternal and fetal health. Ultra-processed foods (UPFs) are increasingly prevalent in pregnancy groups despite being nutritionally unbalanced and associated with adverse perinatal outcomes. This cross-sectional study, conducted with data from 229 pregnant women, aimed to investigate the association between UPFs consumption and dietary nutrient intake of pregnant women assisted by Primary Health Care (PHC) in Federal District (DF), Brazil. Food consumption was assessed through two non-consecutive 24-h food records and categorized by the extent of processing using the NOVA classification. Multivariate linear regression models were used to analyze the association between the quintiles of UPF consumption and the total energy and nutrients intake. Mean daily energy intake was 1741 kcal, with 22.6% derived from UPFs. Greater UPF consumption was associated with reduced intake of unprocessed and minimally processed food. The highest quintile of UPFs was positively associated with higher total energy, trans fat, and sodium intake; and inversely associated with the diet content of protein, fiber, iron, magnesium, potassium, copper, zinc, selenium, and folate. Greater UPFs intake negatively impacts the nutritional quality of the diet and impoverishes the nutrient intake of pregnant women. Reducing UPF consumption may broadly improve dietary guidelines adherence in pregnant women and promote maternal and neonatal health.

## 1. Introduction

During pregnancy, women undergo significant metabolic and physiological changes that include increased nutritional needs in order to achieve proper fetal growth and normal development [1]. It has been well-established that pregnancy increases energy requirements and a woman’s need for protein, vitamins, and minerals [2], especially iron, zinc, calcium, iodine, vitamin D, folate, and other B vitamins [3]. 

Adequate quality of diet and nutritional status during pregnancy are crucial to optimize maternal and fetal health and promote successful pregnancy outcomes [4]. On the other hand, poor maternal diet, lacking in key nutrients, is associated with adverse perinatal outcomes such as maternal anemia, pre-eclampsia, hemorrhage, and increased risk of maternal mortality. It can also lead to stillbirth, inadequate birthweight, miscarriage, and fetal development complications [5]. Thus, it is fundamental to identify modifiable dietary risk factors related to such adverse outcomes of pregnancy. 

Higher consumption of diets rich in ultra-processed foods (UPFs) during pregnancy was found to be associated with an increased risk of gestational diabetes mellitus and preeclampsia [6]. Despite the evidence of the relationship between maternal diet and perinatal outcomes, several studies have reported inadequate diet quality during pregnancy, including high consumption of unhealthy foods and UPFs [7,8,9,10].

UPFs are one group, in the four-category NOVA classification system, that categorize foods according to the extent and purpose of processing: unprocessed or minimally processed, processed culinary ingredients, processed food, and UPF. Keding et al. [11] interestingly emphasize the relevant role that food processing is playing in the food system sustainability, specifically regarding a sustainable diet due to the combination of low-cost ingredients at purchase and increased consumption worldwide. Regarding UPFs, they are formulations of ingredients, mostly of industrial use, made from substances extracted from food, with little or no whole food [12]. Most of them are high energy-dense products, rich in sugar, unhealthy fat and salt, and low in dietary fiber, protein, vitamins, and minerals. In addition, the industrial process often includes artificial additives in order to create durable, accessible, convenient, and attractive products. Some examples include soft drinks, processed meats, ice cream, snacks, sweets, instant soups, and fast foods [13].

There is increasing evidence that a high consumption of UPFs negatively affects nutritional dietary quality as is associated with an increase in free sugars, total fats, and saturated fats, as well as a decrease in nutrients such as fiber, protein, potassium, zinc, and magnesium, and vitamins A, C, D, E, B12, and niacin in adult population [14,15,16]. A recent systematic review showed that despite the limited literature on UPF consumption and health outcomes in the maternal-child population, the highest UPF consumption negatively impacted nutrition and disease development indicators in pregnant, lactating women and children [17]. The UPF are currently present in the dietary pattern of several high-income countries such as the United States, Canada, and the United Kingdom, and contribute on average greater than half of energy consumed [18]. The 2017–2018 Household Budget Survey showed that UPFs intake represents 19.7% of the daily caloric intake of Brazilians [19]. However, there is still limited data about Brazilian pregnant women’s consumption of UPFs and its impact on nutritional dietary profile and quality of diet. 

Considering the role of maternal diet on the health of the mother–child binomial, it is fundamental to understand the effect of UPF consumption during pregnancy on dietary nutrients intake. Therefore, this study aimed to investigate the association between UPF consumption, based on the NOVA food classification system, and dietary nutrients intake of women assisted by prenatal service in public Primary Health Care (PHC) in Federal District (DF), Brazil.

## 2. Materials and Methods

### 2.1. Study Design and Population

This study was approved by the Ethics Committee of the University of Brasilia (2.977.035) and of the Research Ethics Committee of the Federal District Health Department (3.489.243).

This analytical cross-sectional study is part of the project “Multicenter Study of Iodine Deficiency (EMDI-Brazil)”, conducted with pregnant women assisted by prenatal service in PHC of the Unified Health System (SUS) in Federal District (DF), Brazil. 

For this study, a simple random sample was calculated and performed using the StatCalc tool of the EpiInfo Software (EpiInfo 7.2) (Center for Disease Control and Prevention, Atlanta, GA, USA), considering the average monthly prenatal care appointments at PHC in 2016 as a proxy for the number of pregnant women monitored by PHC in DF (*n* = 18,877—data reported by the DF State Department of Health); and the prevalence of the indicator “consumption of ultra-processed foods the day before” among Brazilian pregnant women monitored by PHC (81.5%), in the same year, from the Food and Nutrition Surveillance System—SISVAN [20]. The acceptable error of 5.5% and the 95% confidence interval (95% CI) were considered. The minimum number of pregnant women to be included was defined as 190; 20% was added to the estimated number, anticipating possible losses. Thus, the sample was estimated at 228 pregnant women. Ten PHC units were selected based on the proximity to the central region and the highest monthly average prenatal care performed in 2016 (data reported by the Distrito Federal State Department of Health).

Pregnant women from all gestational ages (first, second, and third trimesters) who attended the selected PHC units for prenatal follow-up consults were invited to participate before or after the appointment at the units. Participants with hypothyroidism or history of thyroid disease were not included due to possible interference with the primary outcomes investigated by the EMDI-Brazil.

### 2.2. Data Collection and Measures

Data collection was conducted from August 2019 to September 2021, through face-to-face interview. Exceptionally, during the period from November 2020 to April 2021, due to the COVID-19 pandemic restrictions, data collection was carried out via telephone, and a subsequent individual appointment was set for face-to-face application of the food consumption questionnaire.

A semi-structured questionnaire was applied by trained interviewers to obtain socioeconomic and demographic information related to the participant as follow: maternal age, self-reported skin color (white, brown, black, or Asian); maternal education (no education, elementary school, high school, or higher education); paid work in the previous month (yes or no); household income in the previous month (up to USD 94.00; between USD 94.00 and USD 188.00; between USD 188.00 and USD 566.00; above USD 566.00 or not informed); access to government social program (yes or no); cohabitation with a spouse or partner (yes or no) and self-recognition as head of the family (yes or no).

The questionnaire also comprised questions relating to pregnancy health and maternal lifestyle such as: gestational trimester (first, second, or third); previous pregnancies (none, between 1 and 3, between 4 and 6, or more than 6); medical diagnosis of arterial hypertension before pregnancy (yes or no); current use of micronutrient supplement (yes or no); current use of cigarettes (yes or no); and current consumption of alcoholic beverages (yes or no). 

For the current investigation, data regarding pre-gestational weight, last menstrual period data, and current weight and height were collected from the medical records in pregnant women’s prenatal cards. If it was not recorded, self-reported data were collected. The pre-pregnancy BMI was calculated as weight in kilograms divided by height in meters squared and classified as recommended by the World Health Organization (WHO) [21]. 

Gestational weight gain (GWG) was calculated from the difference between the last measured gestational weight and the informed pre-gestational weight and classified as proposed by Kac et al. (2021) for Brazilian women from 10 weeks of gestation [22]. Pregnancy with less than 10 weeks were not considered in the GWG analysis (*n* = 13). For this study, it was considered below the expected GWG < 25th percentile; according to the expected GWG between the 25th percentile and the 75th percentile; and above expected—or excessive—GWG > 75th percentile.

### 2.3. Dietary Assessment

To evaluate participants’ food consumption, a 24-h dietary recall was applied using the 5-step multiple-pass method developed by the United States Department of Agriculture [23]. The Photographic Manual of Food Portion Quantification [24] was used to improve dietary assessment. The manual includes photos of single foods and meals (e.g., mixed rice and beans, lasagna), as well as household measurements (e.g., cups, spoons) and portions of food. A second dietary recall was collected randomly by telephone in 20% of the sample on a nonconsecutive day to correct the within-person variability [25,26,27,28]. Food consumption data from 24-h dietary recall were analyzed for nutrients composition using the Globodiet software, Brazilian version, Data Entry mode, developed by the GloboDiet Initiative, which created and adapted a standardized and computerized method for data collection through of the 24-h Reminder [29]. 

The analysis of food consumption included the assessment of total energy intake, macronutrient intake, and micronutrient intake. The nutritional density of each micronutrient in the diet was expressed in mg or mcg per 1.000 kcal while macronutrients were expressed as the percentage of energy intake (% TEI). Food consumption was categorized according to the processing degree as defined by the NOVA classification: unprocessed or minimally processed, culinary ingredients, processed, and ultra-processed [12]. The exposure variable of interest in this study was the UPF intake. The percentage of relative energy intake from UPFs were distributed into quintiles according to the contribution of ultra-processed foods to the total caloric value of the diet (% kcal). In order to calculate quintiles, participants were ranked according to their UPF energy intake from lowest to highest and then divided into five equal groups. The first quintile was classified as the lowest consumption percentage, and the fifth as the highest percentage of consumption. Micronutrient supplements were not considered in the dietary analysis. 

### 2.4. Data Analysis

The descriptive results were expressed as mean and standard deviation (SD) for continuous variable. To calculate the frequency related to categorical variables, the prevalence was estimated with their respective 95% CI.

The mean share of all NOVA food groups to the total daily energy intake was estimated. The participants were categorized into five strata in accordance with the quintiles of energy shares from UPF consumption. The association between the quintiles of energy from UPF and total energy and nutrients intake was assessed by the multivariate linear regression model. Quintile 1 was the reference category in all regression analyses. All analyses were adjusted for age, years of study, gestational trimester, social welfare program assistance, and work status. The regression coefficients were presented with their respective confidence intervals (95%). A significance level of 0.05 was considered. The statistical analyses were carried out using Stata software 16 (StataCorp. 2019. Stata Statistical Software: Release 16.1. College Station, TX, USA: StataCorp LLC).

## 3. Results

Data from 229 pregnant women enrolled in prenatal care in PHC were included. The participants’ age ranged from 16 to 50 years old (mean age 28 ± 6.2 years). There was only one pregnant woman at the age of 50 in our sample. The majority of women self-reported brown skin color (60.6%) and 10–12 years of study (53.3%), and 44% of them live with a monthly family income of US$188 to US$566. About 15% are assisted by government social welfare program, and 78% reported living with a partner. Planned pregnancy was reported by 37%, and 86% had at least one prenatal visit prior to 13 weeks of pregnancy. Regarding nutritional status, most of them (51.9%) entered pregnancy within a normal BMI range; however, more than 50% presented inadequate GWG for gestational age (below or above the expected). Detailed subject characteristics are presented in Table 1.

The mean daily energy intake was 1741 ± 646 kcal. On average, pregnant women consumed 64.3 ± 18.2% of total energy from unprocessed or minimally processed food, 4.5 ± 4.3% from culinary ingredients, 8.6 ± 9.9% from processed food, and 22.6 ± 17.2% from UPF.

The mean contribution of UPFs to the total energy intake ranged from 2.7%, in the lowest quintile, to 49.9%, in the highest quintile. On adjusted multivariate linear regression, the highest quintile of energy contribution from UPFs was associated with lower intake of calories from unprocessed and minimally processed food (β = −41.6; 95% CI: −46.70, −36.60) and from culinary ingredients (β = −2.41; 95% CI: −4.19, −0.63) compared to the first quintile. Table 2 presents mean percentage of energy intake from each food group during pregnancy according to quintiles of energy intake from UPFs.

Intake of macronutrients is expressed as a percentage of the total energy intake (TEI). Carbohydrates contributed 50% and protein 17% of TEI. Total fat contributed 33%, of which 11.2% was saturated, 10% monounsaturated, 8% polyunsaturated, and 0.78% was from trans fat. Fiber and micronutrients intake are described in Table 3.

Multivariate linear regression analyses (Table 3) indicated that the highest quintile of UPF consumption was significantly and positively associated with an increase of 489 kcal in mean total energy intake (β = 489.0; 95% CI: 218.61, 759.48) and an increase of 0.5% in the contribution of trans fat in TEI (β = 0.5; 95% CI: 0.26, 0.73) comparing to the lowest reference quintile. In contrast, an inverse relationship was observed for protein intake, with a reduction of 5.97% in TEI (β = −5.97; 95% CI: −8.24, −3.70), and dietary fiber, with a reduction of 4.79 g/1000 kcal (β = −4.79; 95% CI: −6.65, −2.93) in the group with higher UPF consumption. No association was observed for carbohydrate and lipids intake.

Regarding micronutrients intake, the analysis showed significantly inverse association between the highest quintile of UPF consumption and intake of dietary iron, magnesium, potassium, copper, zinc, selenium, and folate. As compared with the first quintile of UPF consumption, pregnant women in the highest quintile consumed 30% more sodium, and approximately 36% less zinc, 67% less selenium, 19.5% less iron, and 28% less folate. No significant association was observed for calcium, iodine, vitamin D, A, C, E, and B12 intake. Table 3 describes the analyses of the association between quintiles of UPF consumption and mean energy and dietary nutrient content.

## 4. Discussion

The present study analyzed the nutritional dietary profile, according to UPF consumption, of pregnant women assisted in prenatal public service PHC units. It is well-established that maternal diet quality and nutritional status during pregnancy directly impact maternal and child health [30,31,32,33,34,35]. In addition, there is growing evidence that UPF consumption is related to lower dietary quality in children and adults [14,36,37], and in pregnant women [38,39]. This is the first study to evaluate the impact of UPFs on the nutrient content of the diet consumed by pregnant women in the capital of Brazil.

In Brazil, the Household Budget Survey has shown that the contribution of UPFs to total energy intake increased from 14.3% in 2002–2003 to 19.7% in 2017–2018. Over the last two decades, the contribution of UPF to the total energy intake of the Brazilian population has continuously increased by replacing fresh foods and culinary preparations for ready-to-eat and processed foods [19]. The results from our study showed that UPFs were considerably present in the diet of the evaluated pregnant women. It accounted for 22.6% of total energy intake in this sample, similar to the general Brazilian population [19]. Interestingly, a cross-sectional study conducted with Brazilian pregnant women from the Brazilian Northeast region observed similar UPF consumption (22.2%), and also showed an association with reduced consumption of rice, beans, meat, fruits, and vegetables [39]. Likewise, a cohort study of pregnant women from Sao Paulo showed that UPFs were responsible for 25.4% of daily calories [40]. A greater share of UPF in maternal diet is reported in other countries: among pregnant women in Spain, it accounted to 29.7% of daily calories [10]; in Canada, 47.7%; and in the USA, studies have shown that UPFs comprised over half of the energy intake during pregnancy [7,38].

According to Nilson et al. [41], approximately 57,000 premature deaths were estimated as attributable to the consumption of UPFs in Brazil in 2019, highlighting the impact of industrial food processing on preventable deaths. Thus, the high consumption of UPFs during pregnancy has important clinical significance given its negative impactive on maternal and neonatal health. Sartorelli et al. [42] showed that pregnant women with higher UPF intake had a three times higher chance of obesity when compared to women with lower intake of these foods. In another study, an increase in energy intake from UPFs was associated with an increase in gestational weight gain and neonatal adiposity [7]. A recent systematic review found that the consumption of UPF-rich diets during pregnancy was associated with higher risk of gestational diabetes mellitus and preeclampsia [6].

Consistent with previous research in general population samples, the results from our study demonstrated an inverse association of ultra-processed food intake with several indicators of overall diet quality. In addition to the high share of UPFs in the diet, this present study found that a greater intake of UPF consumption was associated with reduced intake of unprocessed or minimally processed foods. This group includes vegetables, fruits, beans, and meat, which provide critical nutrients for pregnancy such as protein, iron, folate, zinc, and vitamins [5]. The inadequate intake of key nutrients during pregnancy, as well as higher energy intake during fetal development, may modify fetal tissues reprogramming related to increased risk of the offspring to the development of future chronic disease [43].

Greater intake of UPFs was positively associated with higher energy, trans fat, and sodium intake in the pregnant women evaluated. In the same direction, a cohort study of pregnant women found consumption of sugar and sodium above the WHO upper limits in the higher UPF consumption group [40]. Typically, during manufacturing, UPFs undergo the addition of ingredients such as sugar, salt, and fats; hence, they tend to be energy dense and contribute to increased energy intake [13]. On this aspect, Hall et al. found an important association between higher intake of UPFs and higher energy intake in a randomized controlled trial with adults [44]. It is imperative to highlight that during pregnancy, adequate maternal energy intake is a critical point of care given that it is strongly associated with GWG and birth weight [45].

In addition, lower fiber and protein intake was observed in the highest quintile of UPF. Our results corroborate the fact that UPFs are energy-dense and high-fat foods, and low in protein and fiber [13]. The absence of an association between UPFs and saturated fat intake may be explained by the reformulation of processed food products by the food industry to avoid saturated fats in their composition [46].

Another interesting finding was an inverse association between greater UPF intake and nutrient intake such as iron, magnesium, potassium, copper, zinc, selenium, and folate. Our findings are corroborated by several previous studies who had demonstrated the association between UPF intake and lower dietary fiber, vitamins, and minerals daily consumption, showing that UPFs negatively impact the quality of the diet [38,39,40,47]. The negative impact of UPFs on nutrient content in pregnant women observed in this study is of critical concern as pregnancy is a period when nutritional requirements are markedly increased [3]. The literature has shown that even when a well-balanced diet is accessible, micronutrient inadequacies during pregnancy are common, due to a global trend switch to low-quality diets, rich in UPFs, which has led to suboptimal intake especially of iron, iodine, folate, vitamin D, and vitamin B12 [31,48,49]. 

During pregnancy, environmental factors including nutrition have a significant impact on health in adult life [31]. In this phase, micronutrients play an important role in programming of postnatal pathophysiology, and inadequate perinatal nutrient status may adversely affect many developmental processes in the fetus with a negative impact on later life [50]. Key nutrients such as zinc, folate, and B vitamins are involved in one-carbon metabolism, which is necessary for cell proliferation, growth, and protein synthesis in the early stages of gestation. Inadequate intake of these nutrients may lead to several adverse outcomes including preeclampsia, preterm birth, gestational diabetes mellitus, intrauterine growth restriction, adverse birth weight, and stillbirth, as well as perinatal, neonatal and maternal mortality [5].

The lower intake of vegetables, fruits, and whole foods suggests that UPFs may be perceived as more palatable or convenient foods. A recent study demonstrated that UPFs were found to be low-cost and nutrient-poor as compared to unprocessed foods [51]. While food prices are a complex concept, the cost of food is an important determinant, especially in lower and middle-incomes countries. In Brazil, UPFs were the most expensive food group in 1995; however, the price of UPFs underwent successive reductions since the year 2000, and it is estimated that in the year 2026, they will be cheaper than unprocessed food [52]. Some studies already presented that higher UPF consumption may threaten all food system dimensions sustainability due to the combination of low-cost ingredients at purchase and increased consumption worldwide, when compared to raw foods [53,54,55].

The findings of this study reveal that although pregnancy is an important period when nutrition plays a fundamental role in maternal and fetal health, it is noted that the dietary pattern of pregnant women evaluated in this study still does not fully meet the dietary recommendations available. The Dietary Guidelines for the Brazilian Population emphasizes the consumption of a diet based on natural or minimally processed food, rich in plant-based foods, and recommends avoiding UPFs [56]. A study conducted by Gomes et al. (2019) showed the reduction of UPF intake in Brazilian pregnant women in the first and second trimester of pregnancy by 4.6 points in the healthy eating and physical activity interventions group by health professionals in the PHC [57]. These findings may help to target specific populational groups for guidance to reduce the UPF consumption and evidence the importance to better understand the influence of ultra-processed food consumption during pregnancy. Educational interventions discouraging the consumption of UPFs rich diets and encouraging a minimally processed diet rich in fruits and vegetables might be an effective way to reduce the share of UPFs in pregnant diets and to increase micronutrients intake. Further studies approaching nutritional interventions targeting the reduction of UPF in perinatal period are needed to clarify its impact on maternal diet quality.

Since the pregnancy period is considered a window of opportunity to improve maternal–child health [58], eating pattern during pregnancy is a critical point that should be addressed during prenatal care. While the results may support nutritional recommendations for this population it has some limitations. First, the cross-sectional design, which prevents causality evaluation. In addition, the self-reported 24-h dietary assessment depends on participant memory, cooperation, and communication abilities, and might contain some errors due to memory biases and over/underreporting, with the added disadvantage that it cannot describe a typical diet. Also, the estimation of the usual intake was not conducted, then it was not possible to estimate intraindividual variance and nutrient intake adequacy. Furthermore, it is limited to generalizing the results, taking into account the heterogeneity of physiological, and emotional status that may have specific dietary influences across the gestational trimesters. Regarding the sample, the study included only low-risk obstetric population in a prenatal public service. However, in Federal District, public Primary Health Care covers 58.72% of the local population [59].

Despite these limitations, it is important to highlight the study strengths. To date, this is the first study on this topic with data from Brazilian pregnant women from the Federal District region; the dietary assessment was applied by well-trained nutrition professionals using the 5-steps multiple pass methodology, in addition to the use of the photographic manual of food portions, to enhance accuracy of dietary recall and to reduce bias in portion quantification; and data analysis was conducted with robust methods. The results of the present study indicate important public health implications, given that higher UPF consumption may worsen the nutritional quality of the diet.

## 5. Conclusions

The results of this present study indicate that greater UPF intake negatively impacts the nutritional quality and nutrient intake of pregnant women. It suggests that nutritional recommendations for this population should focus not only on nutrient amounts but also on the degree of food processing. The consumption of a diet rich in whole foods, protein sources, fruits, and vegetables should be reinforced during prenatal care as a strategy to improve short and long-term maternal and neonatal health.

## Figures and Tables

**Table 1:** Characteristics of pregnant women assisted in Primary Health Care. Federal District, Brazil, 2019–2021.

Characteristics	*n* ^a^	%	CI (95%)
**Age (Years)**			
≤19	13	5.7	3.31; 9.55
20–34	179	78.2	72.31; 83.06
≥35	37	16.1	11.91; 21.53
**Self-reported skin color**			
White	45	20	15.18; 25.66
Black	36	15.9	11.69; 21.32
Yellow	8	3.5	1.77; 6.94
Brown	137	60.6	54.06; 66.81
**Lives with partner**			
Yes	177	78	72.08; 82.91
No	50	22	17.08; 27.91
**Paid job over the past month**			
Yes	122	53.7	47.19; 60.16
No	105	46.3	39.83; 52.80
**Household income over the previous month (USD)**			
Up to 94.00	13	5.8	3.35; 9.67
94.00 to 188.00	21	9.3	6.12; 13.85
188.00 to 566.00	100	44.2	37.87; 50.81
Over 566.00	59	26.1	20.76; 32.25
Not reported	33	14.6	10.55; 19.86
**Family members**			
<4 members	181	81.5	75.84; 86.12
>5 members	41	18.5	13.87; 24.15
**Access to social welfare program**			
Yes	34	15	10.93; 20.35
No	193	85	79.64; 89.06
**Self-reported as head of household**			
Yes	79	34.8	28.85; 41.26
No	148	65.2	58.73; 71.14
**Education (completed years)**			
Up to 9 years	38	16.7	12.40; 22.20
10 to 12 years	121	53.3	46.75; 59.73
Over 13 years	68	30.0	24.32; 36.26
Smoking habit			
Yes	9	4.0	2.06; 7.46
No	218	96.0	92.53; 97.93
**Alcohol consumption**			
yes	27	12.0	8.38; 17.03
No	197	88.0	82.96; 91.61
**Parity**			
Primiparous	83	36.6	30.52; 43.05
Multiparous	144	63.4	56.94; 69.47
**Trimester of pregnancy**			
1st trimester	35	15.3	11.16; 20.57
2nd trimester	91	39.7	33.57; 46.24
3rd trimester	103	45	38.62; 51.50
**Pre-pregnancy BMI (kg/m^2^)**			
Underweight	9	4.3	2.23; 8.05
Normal weight	109	51.9	45.11; 58.62
Overweight	59	28.1	22.40; 34.59
Obese	33	15.7	11.37; 21.31
**Gestational Weight Gain ^b^**			
Below the expected (<p25)	62	29.7	26.83; 36.24
According to the expected (≥p25 e <p75)	99	47.4	40.64; 54.18
Above the expected (≥p75)	48	23.0	17.73; 29.19

^a^ The total was lower for some variables due to missing information; ^b^ Brazilian classification chart (Kac et al., 2021) [22]; BMI: body mass index; CI: confidence interval.

**Table 2:** Distribution (%) of total energy intake according to food groups by quintiles (Q1, Q2, Q3, Q4, Q5) of ultra-processed food consumption for pregnant women assisted in Primary Health Care. Federal District, Brazil, 2019–2021 (*n* = 227).

Food Group	Mean Energy Intake by Quintiles of UPF (% of Total Energy Intake)					β (CI 95%)	*p* ^a^
	Q1	Q2	Q3	Q4	Q5		
Unprocessed or minimally processed foods	82.58	71.73	66.67	59.25	40.79	−41.65 (−46.70, −36.60)	0.000
Culinary ingredients	5.97	5.29	4.40	3.69	3.31	−2.41 (−4.19, −0.63)	0.008
Processed foods	8.73	11.92	9.60	6.70	5.95	−3.23 (−7.41, 0.94)	0.128
Ultra-processed foods ^b^	2.70	11.04	19.31	30.35	49.93	47.30 (45.08, 49.52)	0.000

β: linear regression coefficient; CI: confidence interval; UPF: ultra-processed food. ^a^ Multivariate linear regression adjusted for age, years of study, gestational trimester, social welfare program assistance, and work status. ^b^ Mean and confidence interval (95%) of energy intake from UPF by quintiles: Q1 = 50.9 Kcal (34.0, 67.7); Q2 = 185.9 Kcal (168.7, 203.1); Q3 = 340.9 Kcal (305.8, 376.1); Q4 = 508.1 Kcal (454.1, 562.2); Q5 = 1003.49 Kcal (876.0, 1131).

**Table 3:** Total mean energy and nutrient intake according to quintiles (Q1, Q2, Q3, Q4, Q5) of ultra-processed food consumption of pregnant women assisted in Primary Health Care. Federal District, Brazil, 2019–2021 (*n* = 227).

		Quintiles of UPF Intake (% of Total Energy Intake)					β (CI 95%) ^a^	*p* ^b^
	Total (SD)	Q1	Q2	Q3	Q4	Q5		
Total Energy Intake (kcal/day)	1741 (646.45)	1537.71	1697.83	1792.18	1670.84	2009.48	489.0 (218.61; 759.48)	0.000
**Distribution of total energy intake (%TEI)**								
Carbohydrate	50 (10.5)	50.58	47.46	50.12	49.17	53.17	2.20 (−1.43; 7.25)	0.188
Protein	17 (5.8)	18.66	18.65	18.28	17.50	12.99	−5.97 (−8.24; −3.70)	0.000
Total fat	33 (7.4)	31.72	34.79	32.31	33.55	34.34	2.49 (−0.61; 5.60)	0.115
Saturated fat	11.2 (3.2)	10.64	12.11	10.65	10.91	11.48	0.72 (−0.58; 2.04)	0.275
Trans fat	0.78	0.55	0.8	0.77	0.81	1.00	0.5 (0.26; 0.73)	0.000
Monounsaturated fat	10 (2.96)	10.00	10.85	9.73	10.03	9.85	−0.21 (−1.45; 1.02)	0.735
Polyunsaturated fat	8 (3.10)	7.78	7.98	7.83	8.47	7.83	0.14 (−1.15; 1.43)	0.830
**Nutrients density**								
Fiber (g/1000 kcal)	11.11 (4.8)	13.36	11.99	11.82	9.96	8.34	−4.79 (−6.65; −2.93)	0.000
Iron (mg/1000 kcal)	5.27 (1.4)	5.48	5.93	5.13	5.35	4.46	−1.07 (−1.64; −0.50)	0.000
Calcium (mg/1000 kcal)	329.08 (161.8)	314.45	343.55	338.69	333.48	315.36	−9.73 (−77.57; 58.10)	0.778
Magnesium (mg/1000 kcal)	136.68 (37.5)	159.07	139.64	139.97	132.36	111.78	−47.83 (−62.44; −3.22)	0.000
Potassium (mg/1000 kcal)	1273.63 (414.7)	1471.23	1334.02	1294.20	1210.61	1053.22	−424.79 (−590.46; −259.12)	0.000
Sodium (mg/1000 kcal)	1283.65 (453.2)	1075.79	1263.31	1340.80	1329.03	1412.67	325.99 (139.64; 512.34)	0.001
Copper (mg/1000 kcal)	0.83 (1.1)	0.98	1.04	0.88	0.76	0.49	−0.49 (−0.96; −0.02)	0.040
Iodine (mcg/1000 kcal)	65.77 (31.2)	62.68	66.23	62.21	72.29	65.56	1.91 (−11.44; 15.27)	0.778
Zinc (mg/1000 kcal)	6.02 (2.7)	6.23	7.42	6.22	6.15	4.07	−2.28 (−3.34; −1.23)	0.000
Selenium (mcg/1000 kcal)	30.40 (69.7)	51.51	22.62	31.71	27.16	18.51	−34.36 (−61.62; −7.09)	0.014
Vitamin D (mcg/1000 kcal)	1.96 (1.9)	2.04	2.17	1.89	2.29	1.41	−0.71 (−1.50; 0.07)	0.076
Vitamin A (mcg/1000 kcal)	408.54 (1205)	409.63	662.96	379.13	384.96	206.67	−213.06 (−732.44; 306.31)	0.420
Vitamin C (mg/1000 kcal)	82.53 (112)	85.3	89.04	111.97	79.01	46.63	−36.87 (−81.50; 7.74)	0.105
Vitamin E (mg/1000 kcal)	4.13 (3.9)	4.45	5.52	3.88	3.57	3.24	−1.18 (−2.83; 0.47)	0.161
Vitamin B12 (mcg/1000 kcal)	3.21 (5.37)	3.19	4.72	2.94	3.75	1.45	−1.90 (−4.17; 0.35)	0.098
Folate (mcg/1000 kcal)	191.99 (82.3)	208.34	215.39	198.31	185.37	152.03	−58.27 (−91.79; −24.75)	0.001

β: linear regression coefficient; CI: confidence interval. TEI: total energy intake; UPF: ultra-processed food. ^a^ Quintile 1 was the reference category. ^b^ Multivariate linear regression adjusted for age, years of study, gestational trimester, social welfare program assistance, and work status.

## Data Availability

Data are available on reasonable request. The dataset used to conduct the analyses is available from the corresponding author on reasonable request.
